# A Novel Dynamic Spectrum-Sharing Method for Integrated Wireless Multimedia Sensors and Cognitive Satellite Networks

**DOI:** 10.3390/s18113904

**Published:** 2018-11-12

**Authors:** Chuang Wang, Dongming Bian, Gengxin Zhang, Jian Cheng, Yongqiang Li

**Affiliations:** 1 College of Communications Engineering, Army Engineering University of PLA, Nanjing 210007, China; chjatice@163.com (J.C.); niceme@vip.sina.com (Y.L.); 2 College of Telecommunications and Information Engineering, Nangjing University of Posts and Telecommunications, Nanjing 210003, China; satlab@126.com

**Keywords:** cognitive radio, wireless multimedia sensor networks, spectrum-sharing, multibeam, dynamic frequency allocation

## Abstract

With the growing demand, Wireless Multimedia Sensor Networks (WMSNs) play an increasingly important role, which enhances the capacity of typical Wireless Sensor Networks (WSNs). Additionally, integrating satellite systems into WMSNs brings about the beneficial synergy, especially in rural and sparsely populated areas. However, the available spectrum resource is scarce, which contradicts the high-speed content required for multimedia. Cognitive radio is a promising solution to address the conflict. In this context, we propose a novel spectrum-sharing method for the integrated wireless multimedia sensor and cognitive satellite network based on the dynamic frequency allocation. Specifically, the Low Earth Orbit (LEO) satellite system plays the role of the auxiliary to connect sensor nodes and the remote control host, and it shares the same frequency with the Geostationary Earth Orbit (GEO) system in the downlink. Because the altitudes of GEO and LEO satellites differ greatly, the beam size of GEO is much larger than that of LEO, which provides the opportunity for LEO beam to reuse the frequency that was allocated to the GEO beam. A keep-out region is defined to guarantee the spectral coexistence based on the interference analysis in the worst case. In addition, a dynamic frequency allocation algorithm is presented to deal with the dynamic configuration caused by the satellite motion. Numerical results demonstrate that the dynamic spectrum-sharing method can improve the throughput.

## 1. Introduction

Wireless Sensor Networks (WSNs) play an increasingly important role in battlefield surveillance, environmental monitoring and health monitoring due to the low-power, low-cost, distributed and self-organizing characteristics [1,2,3]. With the increasing quantity and quality of the demand, e.g., road traffic control, smart home and emergency rescue, Wireless Multimedia Sensor Networks (WMSNs) draw much attention. The WMSNs consist of sensor nodes complemented by additional multimedia traffic sources (i.e., computers, cameras, smart phones and cellular phones) and are capable of storing, processing, and retrieving multimedia data such as video, audio, and images [4]. In addition, the satellite systems can be regarded as an important auxiliary to WMSNs and act as the transmission link for the integrated wireless multimedia sensor and satellite networks. Integrating satellite systems to WMSNs brings about a beneficial synergy in support of many scenarios, especially in rural and sparsely populated areas because of the inherent wide coverage and unrestricted geography [5]. Moreover, Low Earth Orbit (LEO) satellite constellation systems are attracting more attention because of almost the same delay as terrestrial communication systems and the revolutionary mass production of satellites [6].

The satellite-based sensor networks have been investigated for various application scenarios [7,8,9,10]. Specifically, the architectures and applications of satellite-based WSNs and the roles of the satellite systems were studied in [4]. The work in [11] addressed four architectures of satellite-based WSNs and analyzed the performance of average end-to-end packet delay, packet loss rate and overall energy consumption. In addition, Reference [12] presented the model to evaluate the capacity of the remote sensor and satellite network and attempted to optimize the integrated wireless sensor and satellite network schedules.

However, with increasingly many satellites launched into space, the available radio spectrum resource becomes scarce, which contradicts the high bandwidth required for satellite-based WMSNs. Currently, the spectrum resource is mainly allocated and coordinated by International Telecommunication Union (ITU) according to the service regions and categories, and the frequency for satellite is depleting [13]. Nevertheless, the spectrum-sharing method emerges as one of the most promising options for future networks, where different communication systems share the same spectrum.

Joseph Mitola first proposed the concept of cognitive radio in 1999 [14]. With the usage of cognitive radio, the spectrum can be shared between heterogeneous systems, which brings an effective method for addressing the spectrum scarcity issue. It plays an important role in many fields of research, e.g., Internet of Things [15], cellular mobile communication [16], WiMAX [17], wireless sensor network [18], aeronautical communication [19], ad hoc network [20] and satellite communication [21]. In addition, many cognitive technologies are presented, e.g., spectrum sensing [22,23,24,25], smart antennas and beamforming [26,27,28], beacon signaling [25,29], interference alignment [30,31,32], precoding [33], power control [34,35,36,37,38], beamhopping [39,40] and network coding [41].

Although cognitive radio in terrestrial wireless systems has been deeply studied, the usage in satellite communications faces new challenges, e.g., system architecture, propagation model, round-trip delay, receiver characteristics, satellite characteristics, broad beam coverage, power level and limited possibilities of evolution due to the long system development and the fixed design space segment [37]. There are many open avenues to exploit cognitive radio in satellite communications, especially for the dual satellite network, in which the spectrum is shared between two satellite systems. In addition, most existing studies focus on the static scenarios, where the satellites are Geostationary Earth Orbit (GEO). There are tricky problems to deal with when the cognitive radio is adopted in Non-GeoStationary Orbit (NGSO) satellites because of the satellite motion. The most critical issue lies in the arising of in-line interference when satellites and users from different systems are in alignment, which makes systems paralyzed [42].

To address this issue, the OneWeb system introduced an innovative technique, i.e., progressive pitch, which avoids the interference by gradually and slightly tilting the satellite as it approaches the equator [6]. Nevertheless, it is well known that adjusting the satellite attitude results in the consumption of fuel whether the propulsion is powered electrically or chemically, which will shorten the lifetime of the satellite. Reference [37] reviewed the database approach and pointed out that the awareness of other systems’ operational characteristics, such as frequency allocations, orbital positions, and antenna patterns, is the key for the successful coexistence between NGSO satellite systems. In this regard, Reference [38] proposed an Adaptive Power Control (APC) technique to ensure the spectral coexistence between the GEO and NGSO satellites. In addition, Reference [40] proposed a cognitive broadband satellite network based on the beamhopping and APC techniques, and the feasibility of the APC was verified by calculating how fast the power adaptation to be performed in [37]. However, the spectral efficiency of the secondary system should be declined to protect the primary system when APC is adopted.

In this paper, we propose a novel spectrum-sharing method for integrated wireless multimedia sensor and cognitive satellite networks based on the dynamic frequency allocation. Specifically, the LEO satellite system functions as the auxiliary to connect the sensor nodes and the remote control host, and the LEO satellite user plays the role of the sink node. The LEO system shares the same spectrum with the incumbent GEO system, where the GEO satellite acts as the primary system, and the LEO satellite constellation serves as the secondary system. Through the interference analysis between the GEO and LEO satellites, a keep-out region is proposed to guarantee the spectral sharing, which is defined as the minimum spatial isolation distance between GEO and LEO beams. When the LEO beam approaches the keep-out region of the GEO co-channel beam, which means that the LEO and GEO beams with the same frequency overlap, the frequency will be reallocated to avoid the Co-Channel Interference (CCI). In addition, a dynamic frequency allocation algorithm is presented, which can simultaneously enhance the spectral efficiency and reduce the switching frequency of the beam frequency during the satellite motion.

The remainder of this paper is organized as follows: Section 2 presents the spectrum-sharing method for the integrated wireless multimedia sensor and cognitive satellite network. The interference analysis and the dynamic frequency allocation algorithm are discussed in Section 3. Section 4 presents the numerical simulation. Section 5 concludes this paper.

## 2. Spectrum-Sharing Method for the Integrated Wireless Multimedia Sensor and Cognitive Satellite Network

**Figure 1:**
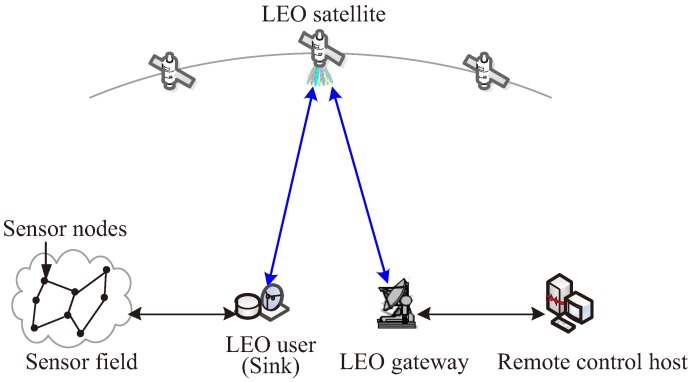
Architecture of the integrated wireless multimedia sensor and cognitive satellite network.

Figure 1 depicts the architecture of the integrated multimedia wireless sensor and cognitive satellite network proposed in this work, which is composed of the sensor nodes, the sink, the LEO satellite system and the remote control host. The LEO satellite system plays the role of the auxiliary to connect the sensor nodes and the remote control host, and it shares the same frequency with the GEO system in the downlink. More details are presented as follows.

Sensor nodes: Sensor nodes in the sensor field sense different dynamic physical quantities and convert the quantities into electrical signals. In addition, some are complemented by additional multimedia traffic sources (i.e., computers, cameras, smart phones and cellular phones). The electrical signals are digitalized and encapsulated into data packets and exchanged with the sink through the terrestrial wireless link.

LEO user (Sink): The LEO user functions as the sink which provides the interface between the satellite system and the terrestrial sensor network. It can store and retrieve multimedia data such as video, audio, and images.

LEO satellite system: The LEO satellite system is regarded as the auxiliary to connect sensor nodes and the network control center. The LEO user exchanges the data with the LEO gateway through the communication link of the satellite, including video, audio, images and other data streams.

Remote control host: Remote control host is connected to the LEO gateway through the Internet or the private terrestrial network. It mainly transmits and receives data packets to send and obtain the information, e.g., multimedia contents and control signalings.

**Figure 2:**
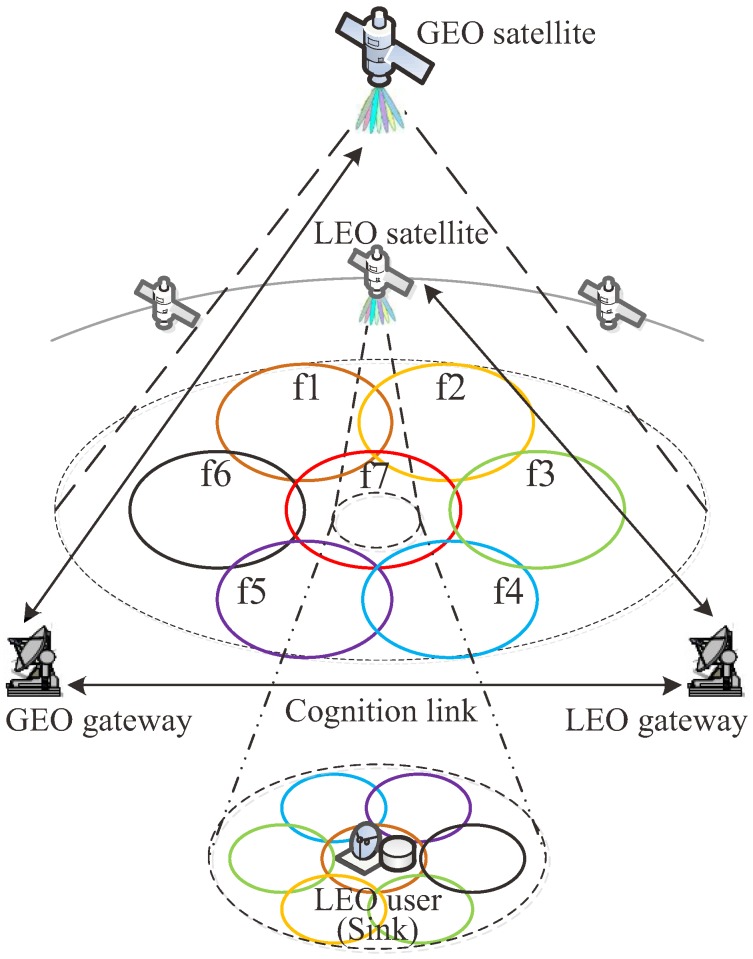
Schematic of the novel spectrum-sharing method.

Figure 2 depicts the schematic of the novel spectrum-sharing method for the integrated multimedia wireless sensor and cognitive satellite network. The primary GEO system is an already deployed system, whereas the secondary LEO system adapts its frequency scheme to ensure the unobstructed operation of the primary system. Both systems provide fixed service with the same spectrum in the downlink, and the antennas of the users keep tracking their corresponding satellites precisely. The two systems deploy multibeam payloads which leverage the spatial reuse of spectrum resource. The frequency scheme of the GEO satellite is static, and the frequency reuse factor is seven [43], whereas those of the LEO satellite varies with the satellite motion. The cognition is achieved by sharing the satellite ephemeris and frequency scheme with the help of the signaling link between the gateways.

The beam size of GEO is much larger than that of LEO because the satellite altitudes differ greatly. An LEO beam that is inside a GEO beam can use any frequencies except for that allocated to the GEO beam. Thus, the frequency division multiplexing can be implemented among the beams of LEO satellite, and the frequency reuse factor is smaller than seven. However, the overlap between GEO and LEO is dynamic because the LEO satellite moves relative to the ground, and the CCI occurs if the co-channel beams cover the same region. The gateways of LEO and GEO systems are connected so that the cognition is achieved by sharing the current frequency scheme and the satellite ephemeris. Therefore, it can be predicted when and where the interference will occur. Once the interference is imminent, the frequency allocation will be adjusted. It should be noted that, with elaborate frequency allocation, the interference can be avoided totally rather than mitigated, which implies a higher spectral efficiency.

## 3. Interference Analysis and Dynamic Frequency Allocation

### 3.1. Interference Analysis Model

We consider Signal to Interference plus Noise Ratio (SINR) to be the signal quality indicator and its formula is:

(1) $$\gamma =\frac{\frac{P_{D}G_{T,D}G_{R,D}}{L_{D}}}{\frac{P_{I}G_{T,I}G_{R,I}}{L_{I}}+kT_{n}B},$$

where *P* represents the transmit power; $G_{T}$ represents the gain of transmit antenna; $G_{R}$ represents the gain of receive antenna; *L* represents the free space path loss; $T_{n}$ represents the equivalent noise temperature of receiver; *B* represents the transponder bandwidth, and *k* is Boltzmann constant. D in subscript denotes the variable related to the desired link whereas I for the interference link. The free space path loss is:

(2) $$L={\left(\frac{4\pi d}{c/f}\right)}^{2},$$

where *f* and *d* represent the frequency and the distance between the transmitter and receiver, respectively, and c is the velocity of light. The expression of the antenna gain is [44]:

(3) $$G=G_{0}{\left(\frac{2J_{1}\left(\mu \right)}{\mu }\right)}^{2},$$

where μ=πfDsinθ/,c; $J_{1}$ is the first order Bessel function; θ is the off-boresight angle of the transmitter or receiver; $G_{0}$ is the maximum antenna gain corresponding to angle zero, and its expression is:

(4) $$G_{0}=\eta {\left(\frac{\pi D}{c/f}\right)}^{2},$$

where *D* represents the antenna diameter, and η represents the antenna efficiency.

It can be inferred that the value of SINR mainly depends on the off-boresight angles and the distances. Due to the LEO motion, the angles and the distances that are determined by the geometrical configuration vary with time. Particularly, the off-boresight angles of the desired receiver are zero because the antennae of GEO and LEO users keep pointing their corresponding satellites.

### 3.2. Interference Analysis in the Worst Case

**Figure 3:**
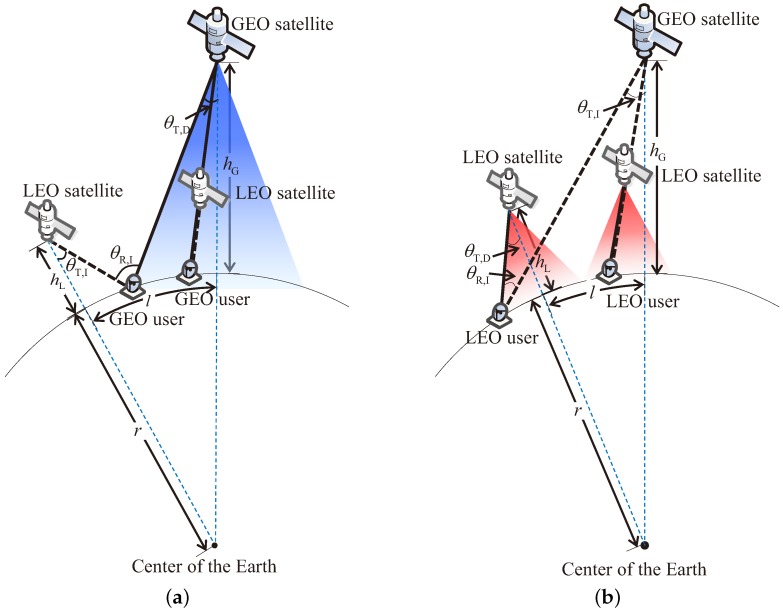
Geometrical configuration in the worst case. (**a**) GEO user; (**b**) LEO user.

When the LEO beam approaches the co-channel beam of the GEO satellite, the CCI becomes strong, and the signal quality of both systems declines accordingly. During the approach, there is a worst-case situation for both GEO and LEO users which means the signal quality is the lowest at every moment. Figure 3a depicts the geometrical configuration of the worst case for the GEO user. The solid line denotes the desired link and the black dashed denotes the interference link. When the LEO satellite is away from the GEO satellite, the worst case occurs if the GEO user locates at the edge of the GEO beam, where the desired link is the weakest, and the interference link is the strongest. Contrarily, when the LEO satellite has moved into the GEO beam, the worst case occurs if the GEO user is on the extension line between the GEO and LEO satellites. Because the quality of the desired link is almost changeless in the desired beam, the situation with the strongest interference link signifies the worst case. The two cases are shown in the left and right sides of Figure 3a, respectively. Based on the geometrical analysis, the off-boresight angles and the distances related to the GEO user can be calculated as follows:

when l>rπ−0.5θG,3dB−arcsinBsin0.5θG,3dBBsin0.5θG,3dBAA,

(5) $$\theta_{T,D}=0.5\theta_{G,3\mathrm{dB}},$$

(6) dD=rsinπ−θT,D−arcsinBsinθT,DBsinθT,Drrrsinπ−θT,D−arcsinBsinθT,DBsinθT,DrrsinθT,DsinθT,D,

(7) dI=A2+r2−2Arcosllrr−π+θT,D+arcsinBsinθT,DBsinθT,Drr,

(8) θT,I=arcsinrsinllrr−π+θT,D+arcsinBsinθT,DBsinθT,Drrrsinllrr−π+θT,D+arcsinBsinθT,DBsinθT,DrrdIdI,

(9) θR,I=llrr+θT,I+θT,D,

(10) $$\theta_{R,D}=0,$$

when l≤rπ−0.5θG,3dB−arcsinBsin0.5θG,3dBBsin0.5θG,3dBAA,

(11) θT,D=arcsinAsinllrrAsinllrrA2+B2−2ABcosllrrA2+B2−2ABcosllrr,

(12) θT,I=arcsinBsinθT,DBsinθT,DAA,

(13) dI=rsinπ−θT,I−arcsinBsinθT,DBsinθT,Drrrsinπ−θT,I−arcsinBsinθT,DBsinθT,DrrsinθT,IsinθT,I,

(14) dD=dI+A2+B2−2ABcosllrr,

(15) $$\theta_{R,D}=\theta_{R,I}=0,$$

where T and R in the subscript denote the transmitter- and receiver-related variables, respectively; D and I denote the desired- and interference-related variables, respectively. $A=r+h_{L}$ and $B=r+h_{G}$, where $h_{L}$ and $h_{G}$ denote the orbital altitudes of the GEO and LEO satellites, respectively; *r* denotes the Earth radius; $\theta_{G,3\mathrm{dB}}$ denotes the angle corresponding to 3dB beamwidth for GEO, and *l* is the distance between the boresight intersections of GEO and LEO beams.

Similar to the GEO user, there are two typical geometrical configurations of the worst case for the LEO user, as shown in Figure 3b. The off-boresight angles and the distances related to the LEO user can be calculated as well:

when l>r0.5θL,3dB−arcsinAsin0.5θL,3dBAsin0.5θL,3dBBB,

(16) $$\theta_{T,D}=0.5\theta_{L,3\mathrm{dB}},$$

(17) dD=rsinarcsinAsinθT,DAsinθT,Drr+θT,DrsinarcsinAsinθT,DAsinθT,Drr+θT,DsinθT,DsinθT,D,

(18) dI=B2+r2−2Brcosllrr+π−θT,D−arcsinAsinθT,DAsinθT,Drr,

(19) θT,I=arcsinrsinllrr−π+θT,D+arcsinBsinθT,DBsinθT,Drrrsinllrr−π+θT,D+arcsinBsinθT,DBsinθT,DrrdIdI,

(20) θR,I=llrr+θT,I−θT,D,

(21) $$\theta_{R,D}=0,$$

when l≤r0.5θL,3dB−arcsinAsin0.5θL,3dBAsin0.5θL,3dBBB,

(22) θT,I=arcsinAsinllrrAsinllrrA2+B2−2ABcosllrrA2+B2−2ABcosllrr,

(23) θT,D=arcsinBsinθT,IBsinθT,IAA,

(24) dD=rsinarcsinAsinθT,DAsinθT,Drr+θT,DrsinarcsinAsinθT,DAsinθT,Drr+θT,DsinθT,DsinθT,D,

(25) dI=dD+A2+B2−2ABcosllrr,

(26) $$\theta_{R,D}=\theta_{R,I}=0.$$

**Figure 4:**
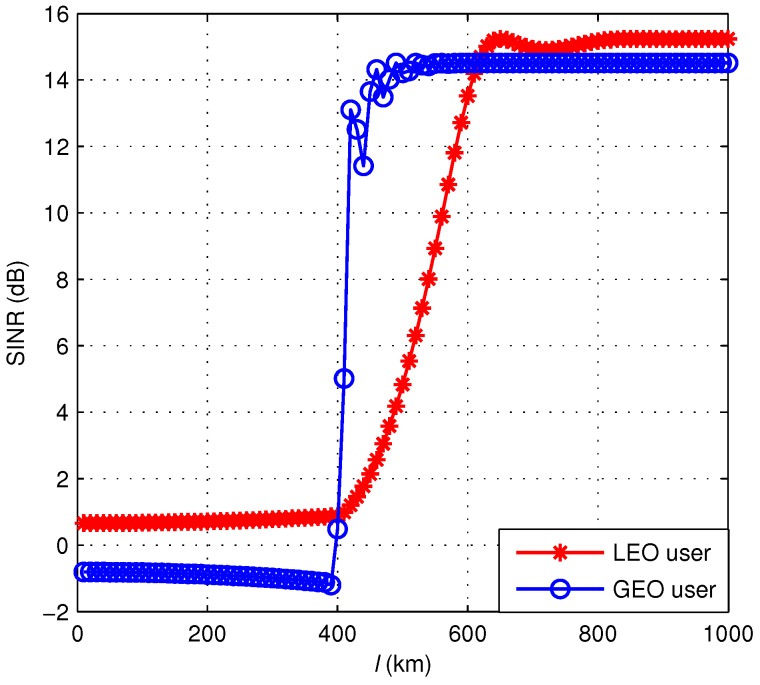
SINR versus *l* for GEO and LEO users.

The satellite orbital parameters and system parameters are presented in Table 1 and Table 2, respectively. Substitute the equations of the angles and distances into Equations (2) and (3) respectively, and then substitute into (1). After a series of calculations, the relationship between the SINR of GEO and LEO users and the distance is shown in Figure 4.

**Figure 5:**
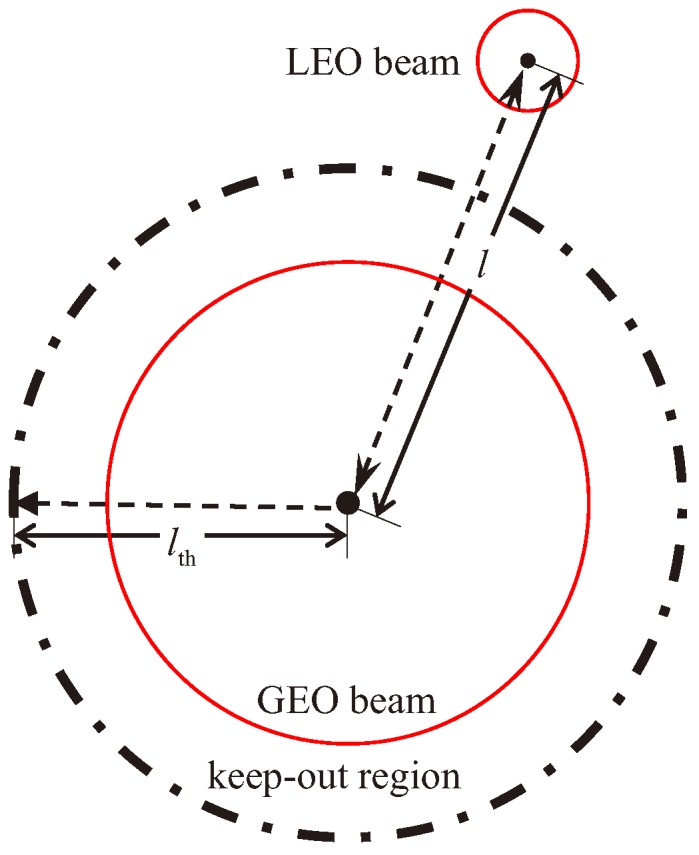
Schematic of the keep-out region.

The numerical result shows that when *l* is smaller than 400 km, which means that the LEO satellite is inside the GEO beam, the CCI is severe, and both systems are paralyzed. When *l* rises, the signal quality rapidly increases until it saturates. It can be inferred that if the co-channel beams from different satellites are far enough away, the interference is almost completely avoided because even the interference in the worst case is very weak. Thus, we consider a keep-out region of the beam to guarantee the normal operation of both systems, whose radius is defined as the minimum spatial isolation distance between GEO and LEO beams, as shown in Figure 5. During the motion of the LEO satellite, spectrum sharing between the two systems can be achieved as long as the LEO beam does not enter the keep-out region of the co-channel GEO beam, i.e., the distance between the boresight intersections of the beams is greater than the radius of the keep-out region.

### 3.3. Dynamic Frequency Allocation Algorithm

We propose a dynamic frequency allocation algorithm based on the keep-out region to achieve the spectral coexistence between the systems. The cognition is achieved through the database approach, i.e., the satellite ephemeris, frequency allocation schemes and antenna patterns are shared between the gateways [37]. The position vector of the satellite in the Earth-Centered Earth-Fixed (ECEF) coordinate can be calculated with the satellite ephemeris [40], and then the position vector of the boresight intersection can be obtained according to the direction of the beam. Eventually, the distance between the two beams is also available. As the already deployed system, the frequency scheme of the GEO satellite is static, whereas the frequency of all LEO beams should be allocated dynamically during the motion of the LEO satellite. If any LEO beam is about to enter the keep-out region of the co-channel GEO beam according to the shared information, the frequency of the LEO beam must be changed. Furthermore, the adjacent LEO beams cannot use the same frequency concerning the frequency division multiplexing in the LEO satellite system. Therefore, all LEO beams need to be considered for reallocation.

Let $x_{ij}\in \left\{0,1\right\}$ indicate whether the *i*-th beam is allocated with the *j*-th frequency, and $d_{ij}$ denotes the distance between the boresight intersections of the *i*-th beam with the *j*-th frequency and the nearest co-channel GEO beam. To minimize the CCI, we consider the maximum total distance of all LEO beams from the corresponding co-channel GEO beams as the objective. The optimization problem of the frequency allocation scheme can be formulated as:

(27) $$\begin{array}{l} \max\sum_{i,j}x_{ij}\cdot d_{ij},\\ s.t.\left\{\begin{array}{l} \sum_{j}x_{ij}=1\left(i=1,\ldots ,N\right),\\ \sum_{j}x_{ij}\cdot d_{ij}\geq l_{\mathrm{th}},\\ x_{ij}+x_{kj}=1\left(j=1,\ldots ,K;i\;\mathrm{and}\;k\;\mathrm{are}\;\mathrm{adjacent},\right) \end{array}\right. \end{array}$$

where *N* represents the number of the LEO beams; *K* represents the frequency reuse factor of the GEO satellite, and $l_{\mathrm{th}}$ represents the radius of the keep-out region.

Usually, the implicit enumeration method is a valid option to solve the 0–1 integer linear programming (27) [45]. However, the computation increases exponentially with *N*. In addition, because of the LEO motion, if it should be optimized in real time, it would be extremely costly, and the beam frequency would switch too frequently. Therefore, we propose Algorithm 1 based on the clustering idea.

**Algorithm 1** Dynamic requency allocation algorithm.**Input:**lth,$T_{0}$ **Output:**$x_{ij}$ **Begin:** Divide LEO beams into $N_{0}$ clusters based on 7-cell frequency reuse pattern. **for**
a=1 to $N_{0}$
**do**  Determine the frequency allocation scheme of each cluster based on (27). **end for** **for**
t=1 to $T_{0}$
**do**  **if**
$\exists i_{0}\in i,s.t.\sum_{j}x_{ij}\cdot d_{ij}<l_{\mathrm{th}}$
**then**   Identify which cluster beam $i_{0}$ belongs to, and redetermine the scheme of this cluster.  **end if** **end for**

**Figure 6:**
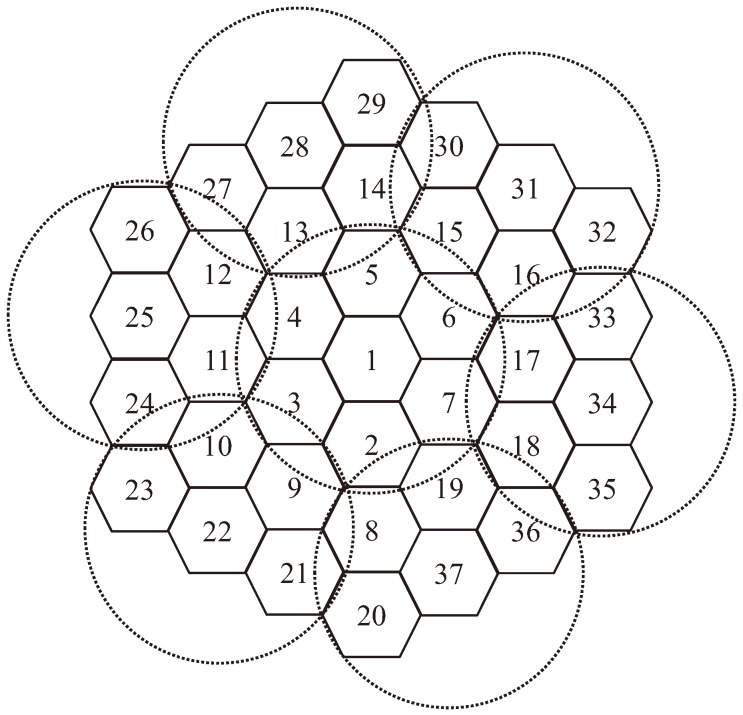
The clustering idea based on a 7-cell frequency reuse pattern.

According to the algorithm, all LEO beams are divided into clusters in Figure 6, and the allocation scheme is optimized in each cluster, which reduces the computation significantly. In addition, the scheme continues until there is an LEO beam about to enter the keep-out region of the co-channel GEO beam, and only the scheme of the conflicted cluster is reallocated, which reduces the frequency of switching.

## 4. Coexistence Simulations

**Figure 7:**
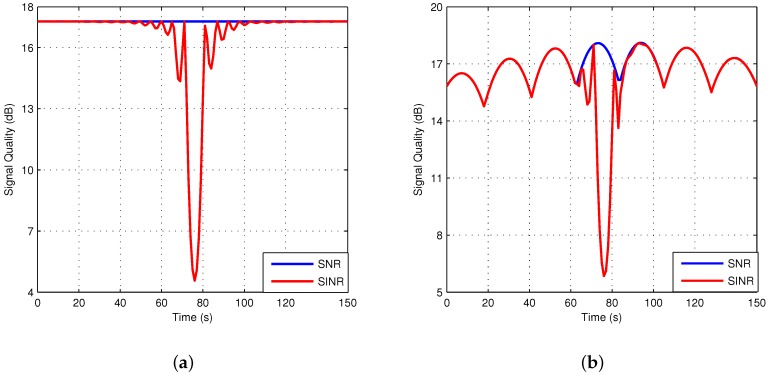
Comparison of signal quality with and without interference. (**a**) GEO user; (**b**) LEO user.

For simplicity, both GEO and LEO users are placed in the same location of the GEO central beam, and the simulation time is set to the duration that the LEO satellite passes over the users. The simulation parameters are presented in Table 3. First, the CCI between GEO and LEO systems is analyzed. Figure 7a,b shows the signal quality of the GEO and LEO users during the simulation, respectively, where the blue curve represents the original signal without interference, and the red curve represents the signal with interference. The original signal quality of the GEO user is almost unchanged because of the static characteristics of GEO satellites, whereas the original signal quality of the LEO user periodically varies as the beams pass through one by one. When the LEO and GEO systems share the frequency, the signal quality in the red curve declines because of the CCI, and it varies with the motion of the LEO satellite. When the satellites and users are in alignment, the signal quality deteriorates severely, which may make systems paralyzed.

**Figure 8:**
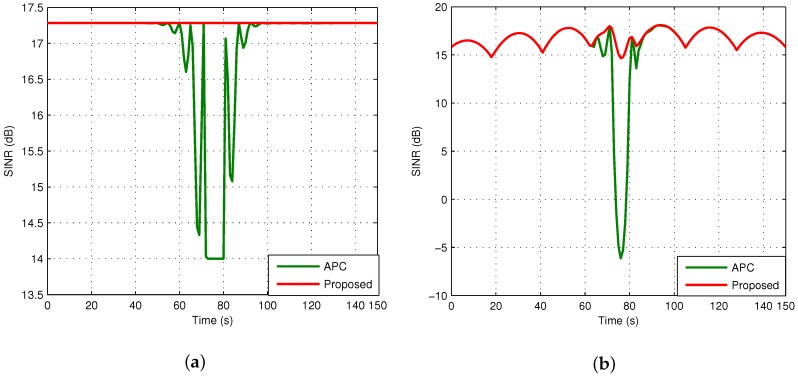
Comparison of SINR between the APC and the proposed algorithm. (**a**) GEO user; (**b**) LEO user.

Next, we analyze the performance of the proposed dynamic frequency allocation algorithm. lth is set to 600 km according to the relationship between the SINR and the distance in Figure 4. During the passage of the LEO satellite, the amount of frequency allocations on clusters is 9, and the total switch of beams is 50, which is much less than frequency allocation without clusters. In addition, the SINR of the proposed algorithm is compared with APC in Figure 8. If the APC is adopted, the SINR of the LEO user declines again because of the protection mechanism for the primary user, as depicted in the green curve. The red curve shows that the SINR of both users for the proposed algorithm performs better than the APC. It should be noted that the SINR of the secondary user is always higher than the threshold, which indicates that the interference can be effectively avoided:

(28) $$ℜ=B\log_{2}\left(1+\gamma_{L}\right)+B\log_{2}\left(1+\gamma_{G}\right).$$

**Figure 9:**
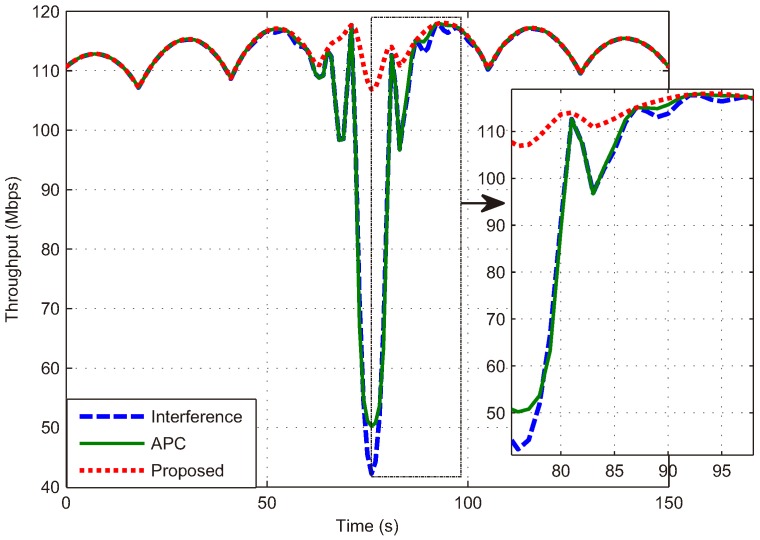
Throughput of the entire network during the satellite passage.

Finally, the total throughput under ideal conditions calculated by (28) is shown in Figure 9, where the blue dashed curve denotes the coexistent systems with interference, and the green solid and red dotted curves denote the networks which employ the APC technique and the proposed algorithm, respectively. We can see that, during the passage of the LEO satellite, when none of the LEO beams enter the keep-out region of the co-channel GEO beams, the two satellite systems can coexist peacefully without protective measures, as shown in the coincidence of curves. When there is an LEO beam entering the keep-out region, the throughput in the blue dashed curve declines because of the in-line interference. The throughput with the APC technique is improved compared to the scenario with interference. However, the improvement is limited because the APC technique guarantees the primary system at the sacrifice of the secondary system. Furthermore, the proposed dynamic frequency algorithm achieves the best performance, twice as large as the others when the in-line interference occurs because the interference can be almost avoided rather than mitigated.

## 5. Conclusions

In this paper, we propose a novel spectrum-sharing method for the integrated wireless multimedia sensor and cognitive satellite network based on the dynamic frequency allocation. The LEO satellite system which shares the same spectrum with the incumbent GEO system functions as the auxiliary to the WMSN. Specifically, the LEO satellite user plays the role of the sink for the terrestrial sensor network. To avoid the co-channel interference, the keep-out region is presented. Furthermore, the dynamic frequency allocation algorithm is proposed. Numerical results evaluate the performance and compare it with the commonly used technique adaptive power control. Allowing frequency reuse between dual satellite systems, the integrated multimedia wireless sensor and cognitive satellite network can enhance the existing WSN applications. The proposed spectrum-sharing method is a potential solution to deliver high-speed content for satellite-based WMSNs with regard to the scarce spectrum. 

## Figures and Tables

**Table 1:** Orbital parameters of the GEO and LEO satellites.

Parameters	GEO	LEO
Semimajor axis	42,164.1 km	7378.14 km
Eccentricity	0	0
Inclination angle	$0^{\circ }$	$90^{\circ }$
Right ascension of the ascending node	$57^{\circ }$	$277^{\circ }$
Argument of perigee	$0^{\circ }$	$0^{\circ }$
Time past perigee	0 s	0 s

**Table 2:** Parameters of the GEO and LEO systems.

Parameters	Notations	Value
Frequency band	*f*	20 GHz (Ka)
Noise temperature of receive antenna	$T_{n}$	290 K
Antenna efficiency	η	55%
Antenna diameter of the user	$D_{U}$	0.4 m
Bandwidth of each beam	*B*	10 MHz
Antenna diameter of GEO satellite	$D_{G}$	0.6 m
Transmit power of GEO beam	$P_{G}$	60 W
Antenna diameter of LEO satellite	$D_{L}$	0.1 m
Transmit power of LEO beam	$P_{L}$	2 W

**Table 3:** Simulation parameters.

Parameters	Notations	Value
Analysis start time	$t_{\mathrm{st}}$	24 August 2018 06:50:51
Analysis stop time	$t_{\mathrm{sp}}$	24 August 2018 06:53:45
Time step	Δt	1 s
Latitude of users	$P_{\mathrm{lat}}$	$1^{\circ }$
Longitude of users	$P_{\mathrm{lon}}$	$25^{\circ }$
Number of LEO clusters	$N_{0}$	7
Frequency reuse factor of GEO satellite	*K*	7
Number of LEO beams	*N*	37
Threshold value of SINR	$\gamma_{\mathrm{th}}$	14 dB

## Abbreviations

The following abbreviations are used in this manuscript:

APC	Adaptive Power Control
CCI	Co-Channel Interference
ECEF	Earth-Centered Earth-Fixed
GEO	Geostationary Earth Orbit
ITU	International Telecommunication Union
LEO	Low Earth Orbit
NGSO	Non-GeoStationary Orbit
SINR	Signal to Interference plus Noise Ratio
SNR	Signal to Noise Ratio
WMSN	Wireless Multimedia Sensor Network
WSN	Wireless Sensor Network

## References

[1] Akyildiz I.F., Su W., Sankarasubramaniam Y., Cayirci E. (2002). A survey on sensor networks. IEEE Commun. Mag..

[2] Aulov O., Halem M. (2012). Human sensor networks for improved modeling of natural disasters. Proc. IEEE.

[3] Astapov S., Preden J.S., Ehala J., Riid A. Object detection for military surveillance using distributed multimodal smart sensors. Proceedings of the International Conference on Digital Signal Processing (DSP).

[4] Celandroni N., Ferro E., Gotta A. (2013). A survey of architectures and scenarios in satellite-based wireless sensor networks: System design aspects. Int. J. Satell. Commun. Netw..

[5] Rawat P., Singh K.D., Chaouchi H., Bonnin J.M. (2014). Wireless sensor networks: A survey on recent developments and potential synergies. J. Supercomput..

[6] Hanson W.A. (2016). In Their Own Words: OneWeb’s Internet Constellation as Described in Their FCC Form 312 Application. New Sp..

[7] Li H., Yin H., Gong X., Dong F., Ren B., He Y., Wang J. (2016). Performance Analysis of Integrated Wireless Sensor and Multibeam Satellite Networks Under Terrestrial Interference. Sensors.

[8] Bisio I., Marchese M. (2007). Satellite earth station (SeS) selection method for satellite-based sensor networks. IEEE Commun. Lett..

[9] Dong F., Li M., Gong X., Li H., Gao F. (2015). Diversity Performance Analysis on Multiple HAP Networks. Sensors.

[10] Wen-Qin W., Dingde J. (2014). Integrated Wireless Sensor Systems via Near-Space and Satellite Platforms: A Review. IEEE Sens. J..

[11] Verma S., Pillai P., Hu Y. Performance evaluation of alternative network architectures for sensor-satellite integrated networks. Proceedings of the 27th International Conference on Advanced Information Networking and Applications Workshops (WAINA’13).

[12] Zhang W., Zhang G., Dong F., Xie Z., Bian D. (2015). Capacity model and constraints analysis for integrated remote wireless sensor and satellite network in emergency scenarios. Sensors.

[13] Clegg A., Weisshaar A. (2014). Future radio spectrum access [Scanning the Issue]. Proc. IEEE.

[14] Mitola J., Maguire G.Q. (1999). Cognitive radio: making software radios more personal. IEEE Pers. Commun..

[15] Nitti M., Murroni M., Fadda M., Atzori L. (2016). Exploiting Social Internet of Things Features in Cognitive Radio. IEEE Access.

[16] Gupta A., Jha R.K. (2015). A Survey of 5G Network: Architecture and Emerging Technologies. IEEE Access.

[17] Ghahremani S., Khokhar R.H., Noor R.M., Naebi A., Kheyrihassankandi J. On QoS routing in Mobile WiMAX cognitive radio networks. Proceedings of the 2012 International Conference on Computer and Communication Engineering (ICCCE).

[18] Kumar S., Hegde R.M. (2015). An Efficient Compartmental Model for Real-Time Node Tracking Over Cognitive Wireless Sensor Networks. IEEE Trans. Signal Process..

[19] Jacob P., Sirigina R.P., Madhukumar A.S., Prasad V.A. (2016). Cognitive Radio for Aeronautical Communications: A Survey. IEEE Access.

[20] Teng Y., Song M. (2017). Cross-Layer Optimization and Protocol Analysis for Cognitive Ad Hoc Communications. IEEE Access.

[21] Jia M., Gu X., Guo Q., Xiang W., Zhang N. (2016). Broadband Hybrid Satellite-Terrestrial Communication Systems Based on Cognitive Radio toward 5G. IEEE Wirel. Commun..

[22] Yucek T., Arslan H. (2009). A survey of spectrum sensing algorithms for cognitive radio applications. IEEE Commun. Surv. Tutor..

[23] Clark M.A., Psounis K. (2017). Equal Interference Power Allocation for Efficient Shared Spectrum Resource Scheduling. IEEE Trans. Wirel. Commun..

[24] Jia M., Liu X., Gu X., Guo Q. (2017). Joint cooperative spectrum sensing and channel selection optimization for satellite communication systems based on cognitive radio. Int. J. Satell. Commun. Netw..

[25] Sharma S.K., Bogale T.E., Chatzinotas S., Ottersten B., Le L.B., Wang X. (2015). Cognitive Radio Techniques Under Practical Imperfections: A Survey. IEEE Commun. Surv. Tutor..

[26] Hoyhtya M., Kyrolainen J., Hulkkonen A., Ylitalo J., Roivainen A. Application of cognitive radio techniques to satellite communication. Proceedings of the 2012 IEEE International Symposium on Dynamic Spectrum Access Networks (DYSPAN 2012).

[27] Yuan C., Lin M., Ouyang J., Bu Y. (2015). Beamforming schemes for hybrid satellite-terrestrial cooperative networks. AEU Int. J. Electron. Commun..

[28] Sharma S.K., Chatzinotas S., Ottersten B. Transmit beamforming for spectral coexistence of satellite and terrestrial networks. Proceedings of the IEEE 8th International Conference on Cognitive Radio Oriented Wireless Networks.

[29] Mangold S., Jarosch A., Monney C. Operator Assisted Cognitive Radio and Dynamic Spectrum Assignment with Dual Beacons—Detailed Evaluation. Proceedings of the IEEE 2006 1st International Conference on Communication Systems Software & Middleware.

[30] Rabbachin A., Quek T.Q.S., Hyundong S., Win M.Z. (2011). Cognitive Network Interference. IEEE J. Sel. Areas Commun..

[31] Sharma S.K., Chatzinotas S., Ottersten B., Access O., Sharma S.K., Chatzinotas S., Ottersten B. (2013). Interference alignment for spectral coexistence of heterogeneous networks. EURASIP J. Wirel. Commun. Netw..

[32] Chatzinotas S., Sharma S.K., Ottersten B. (2013). Frequency packing for interference alignment-based cognitive dual satellite systems. IEEE Veh. Technol. Conf..

[33] Christopoulos D., Chatzinotas S., Ottersten B. User scheduling for coordinated dual satellite systems with linear precoding. Proceedings of the IEEE International Conference on Communications.

[34] Lagunas E., Maleki S., Chatzinotas S., Soltanalian M., Pérez-Neira A.I., Oftersten B. Power and rate allocation in cognitive satellite uplink networks. Proceedings of the 2016 IEEE International Conference on Communications (ICC).

[35] Shi S., Li G., An K., Gao B., Zheng G. (2017). Energy-Efficient Optimal Power Allocation in Integrated Wireless Sensor and Cognitive Satellite Terrestrial Networks. Sensors.

[36] Vassaki S., Poulakis M.I., Panagopoulos A.D. (2017). Optimal iSINR-based power control for cognitive satellite terrestrial networks. Trans. Emerg. Telecommun. Technol..

[37] Hoyhtya M., Mammela A., Chen X., Hulkkonen A., Janhunen J., Dunat J.-C., Gardey J. (2017). Database- Assisted Spectrum Sharing in Satellite Communications: A Survey. IEEE Access.

[38] Sharma S.K., Chatzinotas S., Ottersten B. (2016). In-line interference mitigation techniques for spectral coexistence of GEO and NGEO satellites. Int. J. Satell. Commun. Netw..

[39] Sharma S.K., Chatzinotas S., Ottersten B. (2015). Cognitive beamhopping for spectral coexistence of multibeam satellites. Int. J. Satell. Commun. Netw..

[40] Wang C., Bian D., Shi S., Xu J., Zhang G. (2018). A Novel Cognitive Satellite Network with GEO and LEO Broadband Systems in the Downlink Case. IEEE Access.

[41] Alegre-Godoy R., Vazquez-Castro M.A. (2013). Spatial Diversity with Network Coding for ON/OFF Satellite Channels. IEEE Commun. Lett..

[42] Liolis K., Schlueter G., Krause J., Zimmer F., Combelles L., Grotz J., Chatzinotas S., Evans B., Guidotti A., Tarchi D. Cognitive Radio Scenarios for Satellite Communications: The CoRaSat Approach. Proceedings of the 2013 Future Network & Mobile Summit.

[43] Chandler C., Hoey L. (2002). Advanced Antenna Technology for a Broadband Ka-Band Communication Satellite. Technol. Rev. J..

[44] Maral G., Bousquet M. (2011). Uplink, downlink and overall link performance; intersatellite links. Satellite Communications Systems: Systems, Techniques and Technology.

[45] Goel P. (1981). An Implicit Enumeration Algorithm to Generate Tests for Combinational Logic Circuits. IEEE Trans. Comput..

